# Evaluation of a multi-atlas CT synthesis approach for MRI-only radiotherapy treatment planning

**DOI:** 10.1016/j.ejmp.2017.02.017

**Published:** 2017-03

**Authors:** F. Guerreiro, N. Burgos, A. Dunlop, K. Wong, I. Petkar, C. Nutting, K. Harrington, S. Bhide, K. Newbold, D. Dearnaley, N.M. deSouza, V.A. Morgan, J. McClelland, S. Nill, M.J. Cardoso, S. Ourselin, U. Oelfke, A.C. Knopf

**Affiliations:** a Faculty of Sciences, University of Lisbon, Campo Grande, Portugal; b Division of Radiotherapy and Imaging, The Institute of Cancer Research, London, United Kingdom; c Translational Imaging Group, Centre for Medical Imaging Computing, University College London, London, United Kingdom; d Royal Marsden Hospital, London, United Kingdom; e Centre for Medical Image Computing, Dept. Medical Physics and Biomedical Engineering, University College London, London, United Kingdom

**Keywords:** MRI-only radiotherapy workflow, Synthetic CT, Multi-atlas approach

## Abstract

**Background and purpose:**

Computed tomography (CT) imaging is the current gold standard for radiotherapy treatment planning (RTP). The establishment of a magnetic resonance imaging (MRI) only RTP workflow requires the generation of a synthetic CT (sCT) for dose calculation. This study evaluates the feasibility of using a multi-atlas sCT synthesis approach (sCT_a_) for head and neck and prostate patients.

**Material and methods:**

The multi-atlas method was based on pairs of non-rigidly aligned MR and CT images. The sCT_a_ was obtained by registering the MRI atlases to the patient’s MRI and by fusing the mapped atlases according to morphological similarity to the patient. For comparison, a bulk density assignment approach (sCT_bda_) was also evaluated. The sCT_bda_ was obtained by assigning density values to MRI tissue classes (air, bone and soft-tissue). After evaluating the synthesis accuracy of the sCTs (mean absolute error), sCT-based delineations were geometrically compared to the CT-based delineations. Clinical plans were re-calculated on both sCTs and a dose-volume histogram and a gamma analysis was performed using the CT dose as ground truth.

**Results:**

Results showed that both sCTs were suitable to perform clinical dose calculations with mean dose differences less than 1% for both the planning target volume and the organs at risk. However, only the sCT_a_ provided an accurate and automatic delineation of bone.

**Conclusions:**

Combining MR delineations with our multi-atlas CT synthesis method could enable MRI-only treatment planning and thus improve the dosimetric and geometric accuracy of the treatment, and reduce the number of imaging procedures.

## Highlights

• Establishing MRI-only RTP workflows requires synthetic CTs for dose calculation.
• This study evaluates the feasibility of using a multi-atlas CT synthesis approach.
• The proposed method was validated on head and neck and prostate cancer patients.
• Results showed an accurate bone estimation for future patient positioning.
• Results showed that synthetic CTs are suitable to perform clinical dose calculations.

## 1 Introduction

Cancer treatment with radiotherapy requires information regarding the patient's anatomy, such as the organs and tumour’s location and the tissue attenuation properties necessary for dose calculations. X-ray computed tomography (CT) is the current gold standard for radiotherapy treatment planning (RTP) mainly because CT intensity values expressed in Hounsfield units (HU) can easily be correlated with tissue electron densities. However, because of its limited soft-tissue contrast, CT imaging can prevent precise and reliable tumour location, particularly in regions such as the brain, head and neck (H&N) or prostate. To overcome this limitation, magnetic resonance imaging (MRI) is being integrated into the radiotherapy workflow. By virtue of their excellent soft-tissue contrast, MR images improve the target volume definition [1], [2]. Avoiding radiation during the imaging protocol is also a major advantage.

The acquisition of both CT and MR images of the patient is already part of the clinical workflow for some indications. MR data is used to define the target volume (i.e. the tumour) and CT data to plan the treatment. Image registration is used to define a spatial relationship between the two images allowing any manual contouring from the MRI to be mapped to the planning CT. However, with this approach, the workflow is extremely dependent on the quality of the image registration [3], [4]. The increased cost and workload for clinicians, when using two different image modalities, is also undesirable [5].

Due to these limitations, there is a growing interest in using an MRI-only RTP workflow. However, as no fundamental relationship between MR image intensities and electron density values exists [6], an accurate method to derive CT equivalent information from MR data (referred to as synthetic CT) is required to perform dose calculations. To assess the feasibility of MR-based treatment planning, the first experiments consisted of assigning single bulk densities to tissue classes (such as bone, air and soft-tissue) delineated either from a CT image [7], [8], [9] or manually from an MR image [10], and then comparing the resulting synthetic CT-based plan to the original CT-based plan. For both H&N and prostate target volumes, dosimetric errors were reported to be 1–2% different from the CT-based dose calculation [8], [9], [10]. Korhonen et al. [11] then explored the possibility of assigning subject-specific density values to the bone class by manually segmenting an MR image and converting the MRI intensity values to HUs using a second-order polynomial model. They showed that this technique improved the plan accuracy when compared with single bulk density assignment. Although these studies showed promising results, their use is limited by the manual delineation step, making them non-viable in an online workflow. Automatic delineation is challenging as bone is not easily distinguishable in traditional MR sequences, due to bone’s short T2^∗^ relaxation time. Despite these challenges, bulk density assignment approaches have recently been made available in clinical RTP software platforms, such as the MRCAT [12] by Philips (Philips, Best, Netherlands), and are already used in practice for cone beam CT-based dose calculations [13] and to account for tissue heterogeneities (i.e. presence of metal implants).

Other methods exist to obtain synthetic CT (sCT) images automatically from MR images and many have been applied to RTP. Hsu et al. [14] used a fuzzy c-means algorithm to segment a set of structural MR images into five tissues classes. The sCT was generated by assigning relative attenuation coefficients with weights based on the probability that each class exists at a given location. Jonsson et al. [15] applied the method developed by Johansson et al. [16] where a sCT was obtained from a Gaussian mixture regression model linking the MRI intensity values to the CT HUs. Another family of methods, the atlas-based methods, rely on a single template [17] or a database of MR and CT image pairs [18], [19], [20], [21], [22], [23]. First, a non-rigid registration between the atlas and test subject MR images is performed. Then, the same transformation is applied to the associated CT images and finally, for the multi-atlas methods, the registered CT images are fused to generate the final sCT. The fusion can be obtained by computing the voxelwise median [21], using a probabilistic Bayesian framework [22], an arithmetic mean process or pattern recognition with Gaussian process [23] or a local image similarity measure [18], [19]. Instead of using a database of images, Andreasen et al. [24] employed a dictionary of MR and CT patches. The sCT was predicted by extracting patches from the test subject MRI, running an intensity-based nearest neighbour search in the patch database and fusing the selected CT patches using a similarity-weighted average. Combining segmentation and use of a template database, Siversson et al. [25] proposed a statistical decomposition algorithm to automatically generate sCTs.

The multi-atlas CT synthesis approach evaluated in this work was first developed for brain applications [26], [27] and then extended to H&N cancer [18]. In this paper, we present a thorough validation of the method, not only for H&N but also for patients with prostate cancer. The main difference with most of the other multi-atlas methods [21], [22], [23] is that the fusion of the atlases is based on the local similarity between each atlas and the test subject. The difference with Dowling et al. [19] is that the proposed approach guarantees a good initial alignment between atlas and test subjects due to a robust affine inter-subject registration process, allows the synthesis from multiple MR sequences and refines the synthesis via an iterative process.

In this paper, we assess the feasibility of implementing our multi-atlas approach into clinical MRI-based RTP on both H&N and prostate cancer patients. We evaluate its performance, both in terms of geometric and dosimetric accuracy, against the standard planning done on a planning CT. To set the results in perspective, we also compare its performance against a synthetic sCT obtained via manual bulk density assignment. To our knowledge, this is the first time that a multi-atlas approach has been applied and evaluated for multiple regions, both H&N and prostate sites, in the context of RTP.

## 2 Methods

### 2.1 Data acquisition

Retrospective data from six H&N patients (with oropharyngeal cancer) treated with volumetric arc therapy (VMAT) and fifteen prostate patients treated with fixed-field intensity-modulated therapy (IMRT) were included in this study. Each patient had a planning CT scan (Philips Big Bore CT), a T1- and T2-weighted turbo spin echo MRI (Siemens 1.5T MRI), a CT delineated structure set and a clinically approved treatment plan (Pinnacle^3^, Philips Medical Systems) to a total dose of 65 Gy and 67–74 Gy for H&N and prostate patients, respectively. All patients were imaged on the same day and in the same position – head-first supine - for both MR and CT image sessions. For H&N patients, the same fixation device was used while, for prostate patients, a different couch was used for MR (curved couch) and CT (flat couch) imaging sessions. For all H&N patients, the resolution of both T1- and T2-weighted MR scans was 0.104 × 0.104 × 0.2 cm^3^. For all prostate patients the resolution of T1- and T2-weighted MR scans was 0.164 × 0.164 × 0.5 cm^3^ and 0.146 × 0.146 × 0.5 cm^3^, respectively. For H&N patients, the resolution of the planning CT was 0.117 × 0.117 × 0.2 cm^3^ while, for prostate patients, it was 0.098 × 0.098 × 0.2 cm^3^. All patients included in this study had given consent for their data to be used for research purposes.

**Fig. 1:**
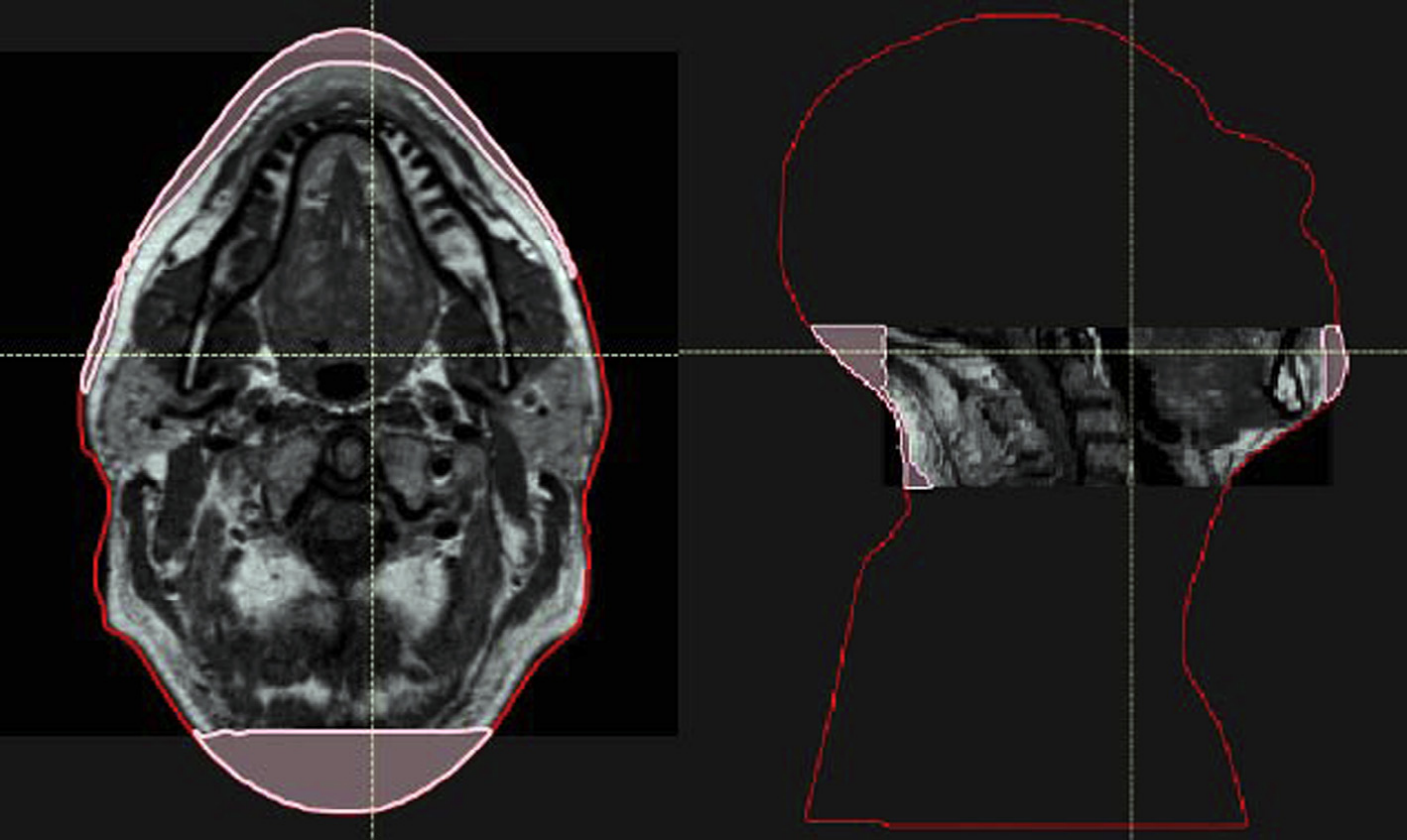
Illustration of the reduced FOV and the lack of MRI coverage for a H&N patient. The original CT outline is represented in red and the missing imaged tissue in pink. Area outside the MRI FOV and inside the red contour was filled-in with a water equivalent density for both the CT and sCTs. Area in pink was assumed to have an air equivalent density for all sCTs. (For interpretation of the references to colour in this figure legend, the reader is referred to the web version of this article.)

**Fig. 2:**
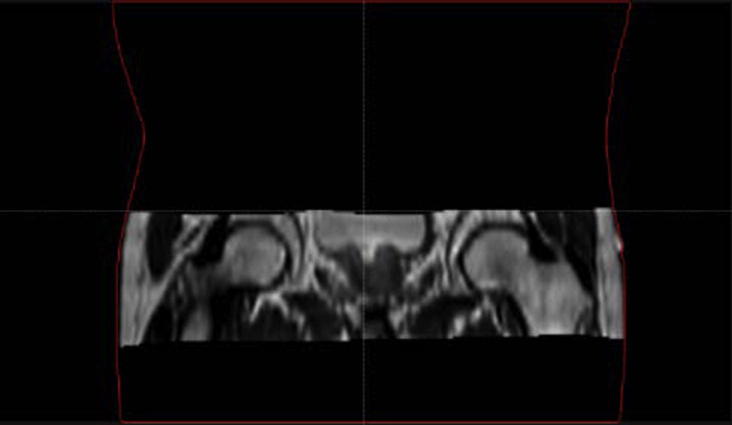
Illustration of the reduced FOV for a prostate patient. The original CT outline is represented in red. Area outside the MRI FOV and inside the red contour was filled-in with a water equivalent density for both CT and sCTs. (For interpretation of the references to colour in this figure legend, the reader is referred to the web version of this article.)

Because of the retrospective nature of the study, inconsistencies exist between the different imaging modalities acquired. A large field-of-view (FOV) was available for the CT scans (scanning level for H&N patients extends from the top of the head to the apex of the lungs and for prostate patients from the abdomen to the lower limbs). In contrast, the MR scans for both H&N and prostate sites where reduced in the cranio-caudal direction, only encompassing the region of interest including the planning treatment volume (PTV) (Fig. 1, Fig. 2). In addition, for the H&N patients, the patient external outline was not fully covered in the MR images, which resulted in missing tissue at the back of the head and on the chin (Fig. 1). Note that this concerns less than 10% of the volume within the MRI FOV.

### 2.2 sCT generation

Two different schemes for the sCT construction were used: the proposed multi-atlas method (sCT_a_) and the manual bulk density assignment (sCT_bda_).

#### 2.2.1 Multi-atlas CT synthesis

The approach for the generation of the sCT_a_ has been described in detail in previous publications [18], [26], [27]. Briefly, the proposed method relies on pre-acquired pairs of non-rigidly registered T2- and/or T1-weighted MR and planning CT images. The non-rigid alignment was necessary to compensate for position differences (and the use of different couches for the prostate patients) between the MRI and CT acquisitions. For the H&N patients, the atlas database was composed of seventeen pairs of T2-weighted MR and CT images, as described in [18], and the method was validated using the images of six other H&N patients not included in the database. Regarding the prostate patients, the atlas database was composed of both T1- and T2-weighted MR, and CT images of fifteen patients. The method was validated following a leave-one-out approach.

To generate the sCT_a_, the first step was to register all the MRIs in the database to the test subject’s MRI. A robust affine registration [18] was used followed by a non-rigid cubic B-Spline registration using normalized mutual information as similarity measure, as implemented in NiftyReg^2^. The robust affine step guarantees that each atlas MR image is well aligned with the test subject despite the large differences in the FOV size and location that were observed between the subjects for both anatomical sites. The transformations were then applied to map the atlas CTs to the test subject MRI. The sCT_a_ was finally obtained by fusing the mapped atlases according to their local similarity to the test subject using a spatial-varying weighted averaging [18].

An iterative process was then used to improve the synthesis [18]. First, the initial sCT_a_ obtained as described above was combined with the test subject MR image(s). Then, all the CT-MR image sets in the atlas database were aligned to the sCT_a_-MR image set using a multi-channel non-rigid registration. The refined sCT_a_ was finally obtained by fusing the registered atlas according to a similarity measure computed between the sCT_a_-MR and mapped CT-MR sets. Combining multiple modalities (MRI and CT) at both the registration and image similarity stages is expected to provide more realistic mappings and improve the local selection of atlases, especially in low contrast areas.

**Fig. 3:**
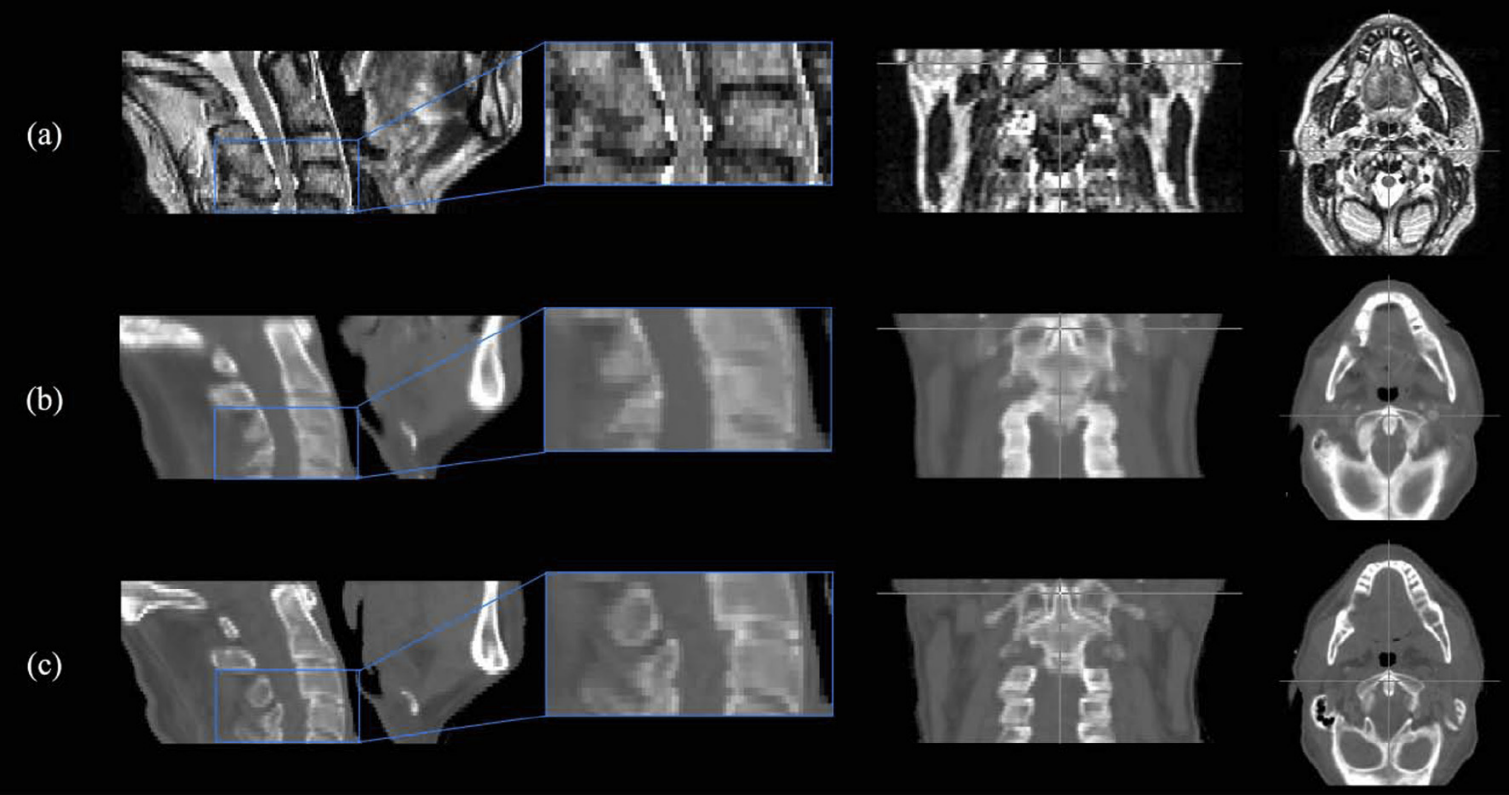
Sagittal, coronal and transverse plane images for a representative H&N patient showing (a) the MRI, (b) the sCT_a_ and (c) the planning CT. MR and sCT_a_ images were non-rigidly aligned to the planning CT for all patients.

**Fig. 4:**
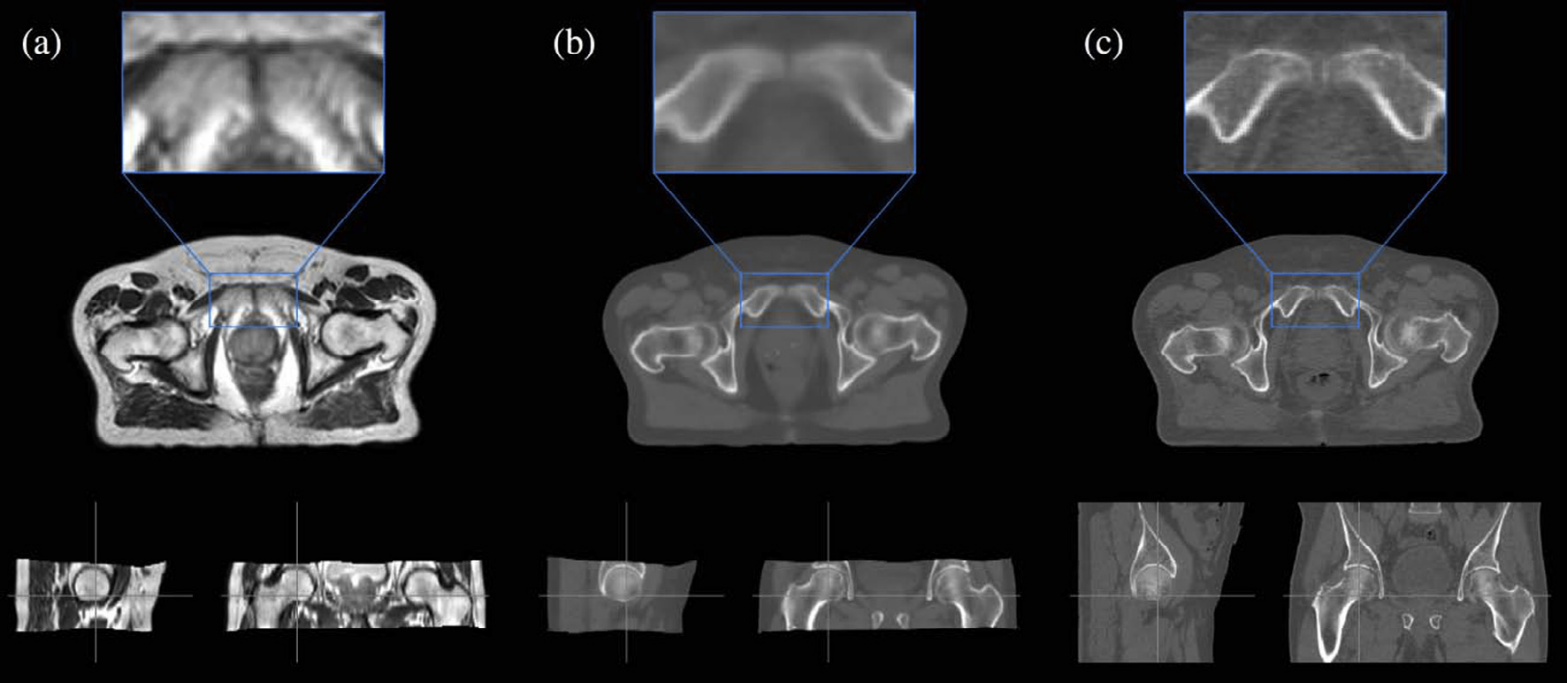
Sagittal, coronal and transverse plane images for a representative prostate patient showing (a) the MRI, (b) the sCT_a_ and (c) the planning CT. MR and sCT_a_ images were non-rigidly aligned to the planning CT for all patients.

As the final step, the sCT_a_ was aligned and resampled to the original planning CT space using the inverse non-rigid transformation mapping the test subject’s CT and MR images, as the registration algorithm chosen is symmetric. This step was necessary to reduce the influence of the different acquisition positions while comparing CT and sCTs [25]. Examples of MR, planning CT and sCT_a_ images are displayed in Fig. 3, Fig. 4.

#### 2.2.2 Manual bulk density assignment

**Fig. 5:**
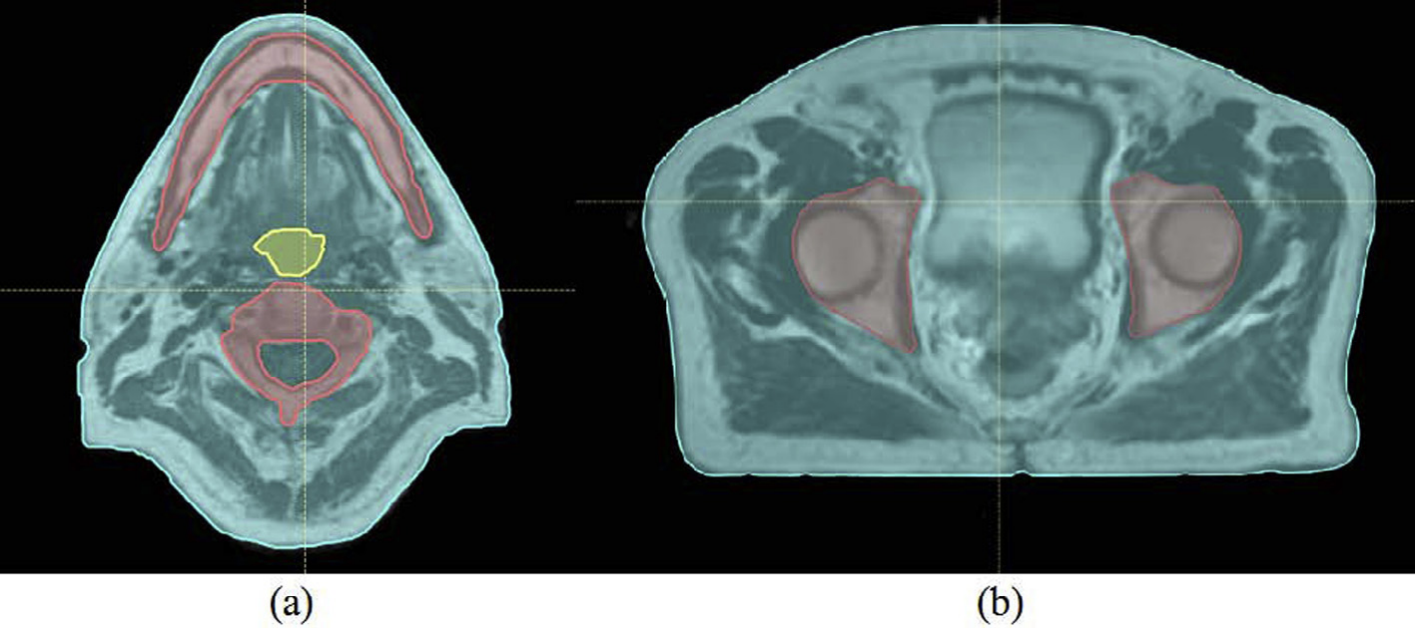
sCT_bda_ obtained for a H&N (a) and a prostate (b) patient. Bone is represented in red, soft-tissue in blue and air in yellow. (For interpretation of the references to colour in this figure legend, the reader is referred to the web version of this article.)

To generate the sCT_bda_ for each patient, the MR scans were non-rigidly registered to the planning CT applying the same transformations used to align the CT and MR images for the sCT_a_ generation. The delineation of the different tissue classes (bone and air), followed by the assignment of specific physical density values to each class, was then carried out using the deformable T2-weighted MR image sets. The rest of the body was defined to be of water equivalent density. For prostate patients, bone (1.22 g/cm^3^) and for H&N patients, bone (1.53 g/cm^3^) and air (0.001 g/cm^3^) tissue classes were defined. Physical densities were defined according to the literature [8], [9], [28]. Bone delineation was performed manually as no efficient threshold exists for bone segmentation using traditional MR sequences. All delineations were done by the same person for consistency and were checked by an experienced physician for adequateness. Air delineation for H&N patients was done using a threshold-based delineation available within the RayStation (Raysearch Laboratories, Stockholm) treatment planning system (TPS). Low MRI intensity values were chosen (<8) and deviations were manually corrected. sCT_bda_ images (Fig. 5) were constructed with the resolution of the original CT image.

### 2.3 Evaluation

The first stage of the evaluation consisted of assessing the accuracy of the generated sCT_a_ and sCT_bda_. Then, the performance of all sCTs against the planning CT was evaluated in terms of geometric and dosimetric accuracy. To reduce the effect of the image discrepancies detailed in Section 2.1, the performance of all sCTs was only evaluated within the FOV where MRI information was available.

#### 2.3.1 Synthetic CT accuracy evaluation

To assess the accuracy of the automatically generated sCTs, the mean absolute error (MAE), defined as

(1) $$\mathrm{MAE}=\frac{1}{N}\overset{N}{\underset{x=1}{\sum }}|\mathrm{sCT}(x)-\mathrm{CT}(x)|$$

was computed for each subject between the sCTs and the planning CT in the external contour, in the bone region and in the soft-tissue region within the MRI FOV. N is the number of voxels x in the considered region. Similarly to Siversson et al. [25], the bone region was defined by thresholding the planning CT at 150 HU within the MRI FOV, and using morphological operators to include softer bone and bone marrow. The soft-tissue region was defined by thresholding the planning CT at −150 HU within the MRI FOV and subtracting the bone region.

#### 2.3.2 Geometric evaluation

The geometric evaluation was performed using the clinical CT contours and delineations on the T2-weigthed MRI (sCT_bda_) and on the atlas-based sCT (sCT_a_). Both external and bone contours within the MRI FOV were evaluated. The external contour was delineated in all images using an automatic threshold tool in RayStation. For the MR images, bone contours were delineated manually while for the CT and sCT_a_ images, the delineations were performed as mentioned before in Section 2.3.1.

The contours were first individually evaluated in terms of shape, position and volume. The contours’ shape and position were visually inspected by overlaying the sCTs’ segmented contours on the CT contours. Changes in volume were evaluated using a volume index (VI) [29]:

(2) $$\mathrm{VI}(A\text{,}B)=\frac{V(A)-V(B)}{V(A)}+1$$

where V(A) is the volume of the clinical CT contour and V(B) the volume of the evaluated contour. VI = 1 indicates identical volumes, while VI > 1 indicates a higher clinical than evaluated contour volume and VI < 1 vice versa.

Finally, an overall evaluation of the contours was performed using the dice similarity coefficient (DSC):

(3) $$\mathrm{DSC}(A\text{,}B)=\frac{2|V(A\cap B)|}{\left|V(A)|+|V(B)\right|}$$

A DSC > 0.7 was considered a good overlap [30].

#### 2.3.3 Dosimetric evaluation

The dosimetric analysis consisted of both gamma (Ɣ) and dose-volume histogram (DVH) analyses. To standardize comparisons between CT and sCTs’ dose distributions, and to check for potential variability in structure definition, the dosimetric accuracy of the generated sCTs was validated using the clinical CT contours. Both organs at risk (OARs) and the target, delineated by a clinician, were rigidly copied from the planning CT to each set of sCTs. Due to the reduced MRI FOV, to simulate the whole body of the patient, the external contour delineated on the planning CT was copied to each sCT and altered in the MRI FOV to be able to maintain the original body outline defined on each sCT. To make a consistent evaluation of the dose distribution differences between image sets, all the regions within the body contour but outside the MRI FOV were assigned to be of water equivalent density in both CT and sCT images (Fig. 1, Fig. 2). For the H&N patients, to maintain the use of the original external contours despite the lack of MRI coverage, the missing tissue in the back of the head and on the chin (Fig. 1) was assumed to be of air equivalent density. For each patient, the original clinical plan was re-calculated using the original planning parameters on the new density override CT and sCTs geometries. Both sCT_bda_ and sCT_a_ were evaluated. All dose calculations were performed using the RayStation TPS with a dose grid of 0.25 × 0.25 × 0.25 cm^3^. Furthermore, in this study the dosimetric influence of the patient couch was of no concern as couch density was set to air in the density override planning CT for the dose re-calculations and it was not present in the sCTs’ image sets.

For the Ɣ-evaluation, a local 3D algorithm implemented in Plastimatch^3^, with constraints of 3% dose difference (DD) and 3 mm distance to agreement (DTA), and 2% DD and 2 mm DTA, using the density override CT dose distribution as reference, was applied. The information from the Ɣ-maps was summarised by calculating the percentage of passing points within the MRI FOV (Ɣ ≤ 1).

DVH metrics including the percentage point difference (PPD) were evaluated for the clinical PTV and OARs cropped within the MRI FOV. The PPD was calculated using the dose value for a specific DVH point in the density override CT dose distribution as the ground truth and the same point in the sCT dose distribution as evaluation. For the target volume D_98%_, D_mean_ and D_2%_ were calculated where D_x_ is the dose given to x% of the structure volume and Dmean is the mean dose given to the evaluated volume. D_98%_ and D_2%_ were used to evaluate the minimum and maximum dose given to the structure, respectively. For the OARs, only D_mean_ and D_2%_ were determined. Spinal cord and right and left parotids for the H&N patients, and rectum and bladder for the prostate patients were evaluated.

## 3 Results

### 3.1 Synthetic CT accuracy evaluation

The average and standard deviation of the MAE obtained between the sCTs and planning CT images are presented in Table 1. We note that the synthesis error is higher for the H&N patients than for the prostate patients.

### 3.2 Geometric evaluation

#### 3.2.1 External contours

**Fig. 6:**
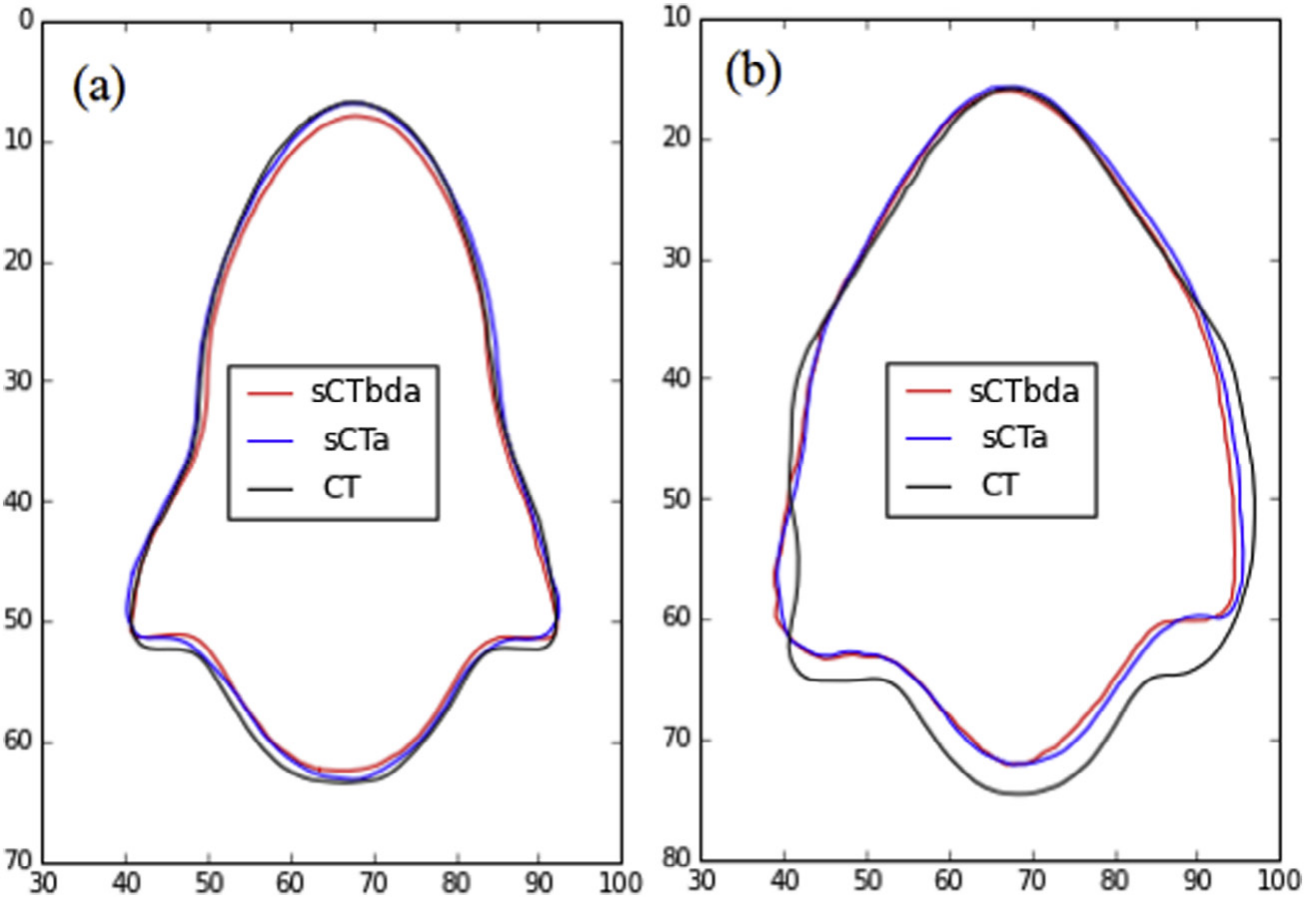
Overlay of CT- (black), sCT_bda_- (red) and sCT_a_- (blue) based delineations for the external contour in (a) a best and (b) worst-case scenario H&N patients. Both sCTs were non-rigidly registered to the planning CT. (For interpretation of the references to colour in this figure legend, the reader is referred to the web version of this article.)

Fig. 6 displays overlays of the external contours for two H&N patients representing the best (Fig. 6 (a)) and worst-case scenario (Fig. 6 (b)).

For the H&N cases, despite the best efforts (same patient positioning and immobilization) small discrepancies in patient positioning and rotation between the CT and MR acquisitions were unavoidable and, for a small number of patients (n = 2), a clear difference in the contours was visible (Fig.6(b)). As a result, these dissimilarities will introduce dosimetric challenges. For the prostate patients, after performing the non-rigid transformation for positioning correction between the CT and MR images, no systematic differences between contours were seen.

**Fig. 7:**
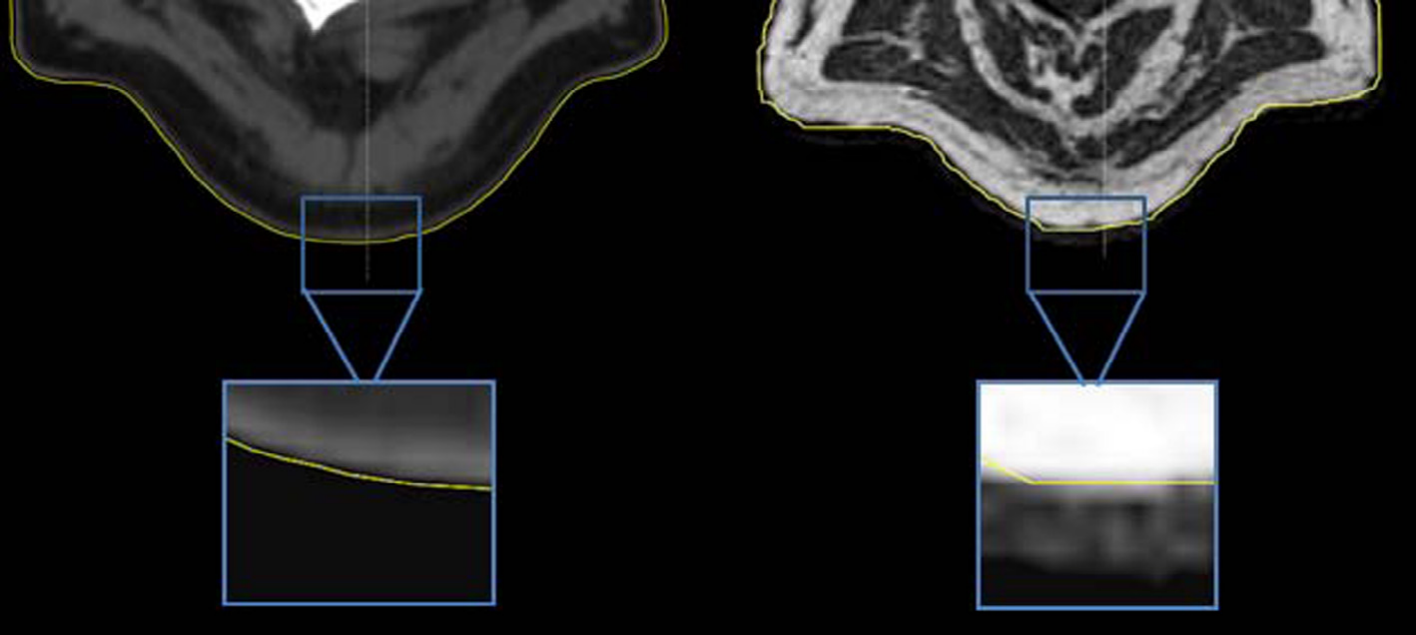
Zoom on CT (left) and on MR (right) images on the patient's boundary. The external contour is represented in yellow. (For interpretation of the references to colour in this figure legend, the reader is referred to the web version of this article.)

In general a good qualitative agreement was observed for the external contour between the images. However, the sCT_bda_-based delineation was systematically a few voxels smaller than the planning CT contour. In Fig. 7, a two-step drop of intensity over a few voxels can be seen in the MR images until the intensity of air outside the patient is reached while for CT images a clear drop is seen. These differences created a systematic difference in the external contours for all patients. When defining the external contour on sCT_a_ images, a higher degree of similarity with the CT contour was observed.

The VI results for the external contours are displayed in Table 2. We can see that the external contour volume is underestimated (VI > 1) for both sCT_bda_- and sCT_a_-based delineations due to the blurry MR boundaries (Fig. 7). Underestimation of the external contour volume is higher for the H&N patients due to the lack of MRI coverage (Fig. 1). However, volumes on sCT_a_ agreed more closely to the original CT volumes.

The DSC values are displayed in Table 3. A high overall similarity with the original contours was achieved for the external contours for all images as the DSC values were larger than 0.7.

#### 3.2.2 Bone contours

**Fig. 8:**
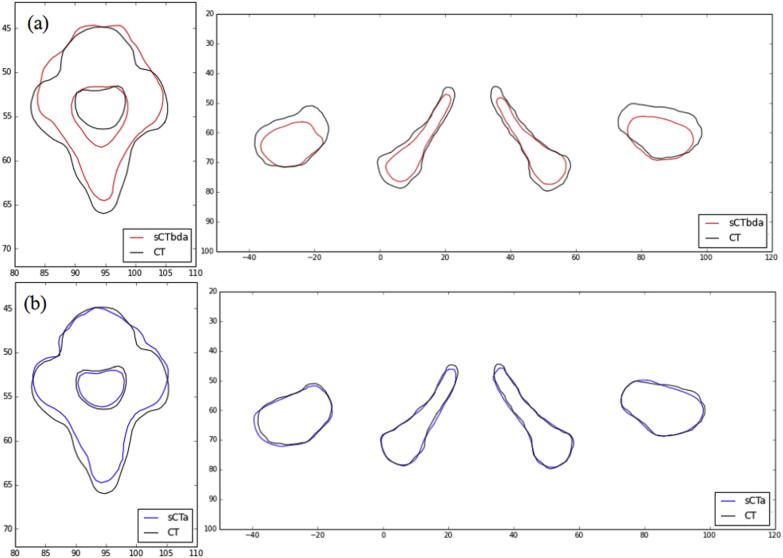
Overlay of (a) sCT_bda_- (red) and (b) sCT_a_- (blue) with CT- (black) based delineations for an H&N and a prostate case. (For interpretation of the references to colour in this figure legend, the reader is referred to the web version of this article.)

Fig. 8 represents the bone contours for a representative H&N and prostate patient. Deviations in shape were observed between the original CT and sCT_bda_ bone contours (Fig. 8 (a)) as a result of the MR-manual delineation and poor bone visibility on the T2-weighted MR sequence. A higher degree of shape similarity was achieved for the sCT_a_ images.

The VI results for the bone contours are displayed in Table 2. A clear trend can be seen for both groups of patients. The sCT_bda_-based contours were smaller (VI > 1) and the sCT_a_-based contours were larger (VI < 1) than the CT-based contours. These differences result from the poor bone visibility on MR and from the blurriness introduced by the atlas method.

The DSC values for the bone contours are displayed in Table 3. As for the external contours, a high overall similarity with the original contours was achieved (DSC > 0.7). However, the high DSC values seen for the multi-atlas approach indicate a closer overall agreement between CT and sCT_a_-based delineations.

### 3.3 Dosimetric evaluation

**Fig. 9:**
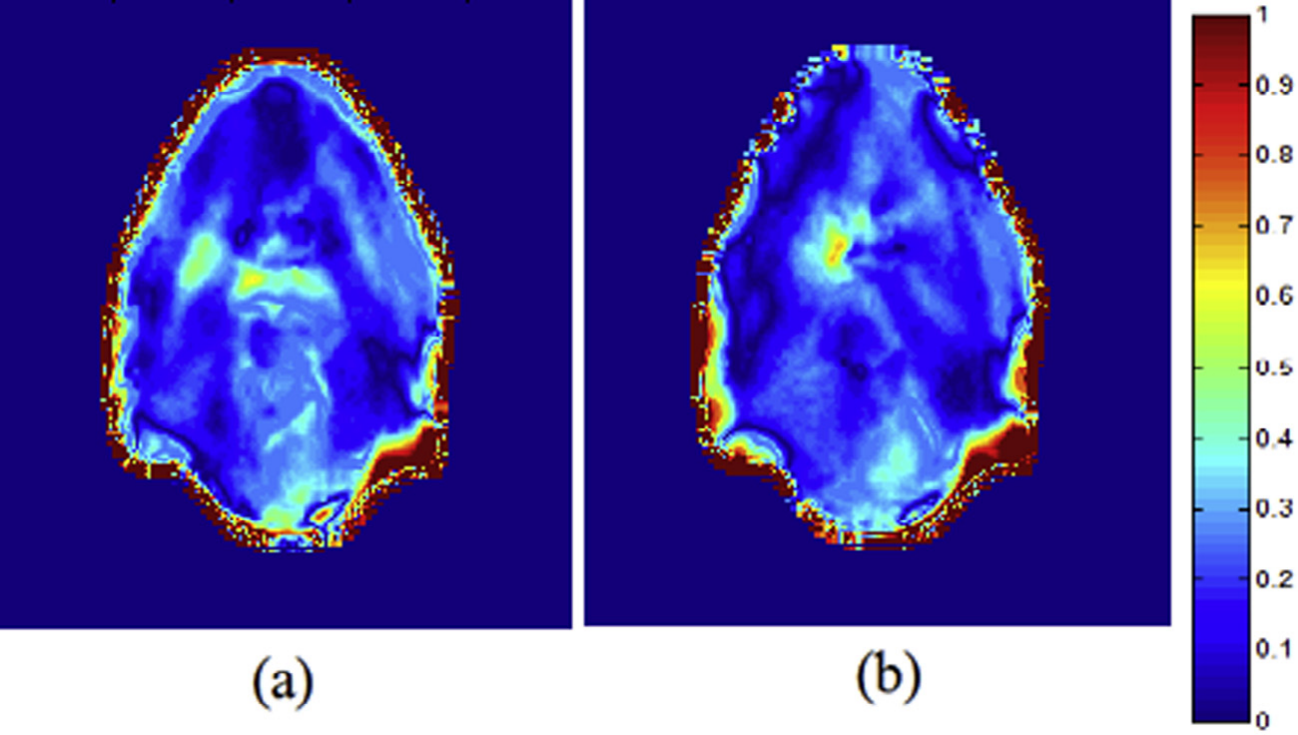
Transversal slice of 3D local gamma maps performed for a combination of 2% DD and 2 mm DTA for (a) sCT_bda_ and (b) sCT_a_ for a representative H&N patient.

**Fig. 10:**
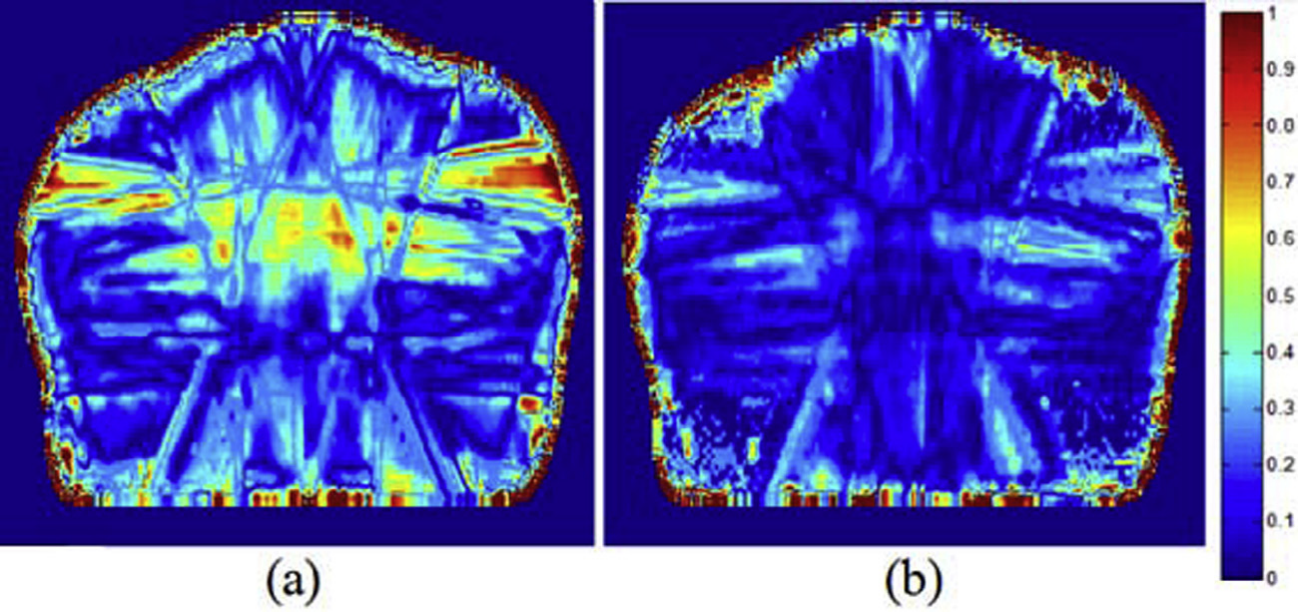
Transversal slices of 3D local gamma maps performed for a combination of 2% DD and 2 mm DTA for (a) sCT_bda,_and (b) sCT_a_ for a representative prostate patient.

Fig. 9, Fig. 10 display gamma maps (2%_2 mm) for representative H&N and prostate patients, respectively. The percentage of passing points for each sCT is detailed in Table 4.

All sCTs displayed a high number of points failing the gamma criteria close to the skin, due to the external contour differences. When using sCT_a_, a greater similarity with the CT dose distribution was observed: a greater number of small gamma values (0–0.3) and a greater number of passing points were obtained when compared to the sCT_bda_ approach.

The PPD between the DVH points from the CT and the generated sCTs are displayed in Table 5, Table 6 for H&N and prostate patients, respectively. Considering all the patients, the mean PPD for the PTV coverage using all sCTs was less than ±0.7% for both the H&N and prostate patients, reaching a maximum individual difference of ±2% of the original dose value. For all evaluated DVH points, patient-specific results were variable.

For the H&N patients, the mean PPD for the OARs was less than ±0.5%, with the maximum individual difference equal to ±1.5%. For the prostate patients, the mean PPD for the OARs DVH points was less than ±0.9%, with the maximum individual difference equal to ±2.0%. For these patients and for the majority of the DVH points analyzed, sCT_a_ tended to have the best agreement with the CT results. However, as verified for the PTV, patient-specific results vary and there is no obvious advantage of using a specific sCT method for dose calculations.

## 4 Discussion

To establish an MRI-only RTP workflow, ensuring accurate dose calculations and geometry delineation from the patient’s MR images is of key importance. This work presents a feasibility study where clinical CT-based dose distributions were compared with those obtained from sCT images generated by our proposed multi-atlas CT synthesis method and by bulk density assignment.

As a first step in the evaluation, we assessed the accuracy of the sCT_a_ obtained with the proposed multi-atlas approach. The MAE obtained within the external contour for the prostate patients was on average 49.8 ± 4.6 HU, which is lower than the error obtained by Kim et al. [20] (74.3 ± 10.9 HU) and is of the same order as the MAE obtained by Siversson et al. [25], Dowling et al. [19] and Andreasen et al. [24] (36.5 ± 4.1 HU, 40.5 ± 8.2 HU and 54 ± 8 HU, respectively), when taking into account the fact that the images used in the present study had a lower resolution. The synthesis error was higher for the H&N patients as the neck is a more challenging area for registration algorithms because of the mixture of bone and air, and due to the presence of large-scale postural changes between patients, such as flexion or extension of the neck and the position of the jawbone.

Then, we carried out a geometric evaluation where the CT-based (used clinically), MR-based (used for sCT_bda_) and sCT_a_-based delineations were compared using the MAE, VI and DSC. The external and bone contours were very similar when delineated on either the original planning CT or sCT_a_. When comparing the sCT_bda_ and CT bone contours, the synthesis error (MAE) was higher, and obvious deviations in shape and in volume were observed. These can be explained by the use of constant HUs for each tissue class to build the sCT_bda_, inter-observer variability, as the delineation was performed manually, and the poor bone visibility in conventional MR sequences. Currently, several groups are working with ultrashort echo time (UTE) sequences to obtain a discriminant signal from bone [16], [31]. In UTE imaging, as the signal is sampled during the free induction decay, before the signal from bone has vanished, it is possible to distinguish bone from air. However, for the MRI-only workflow the number of different MR sequences that can be obtained is limited due to time constraints. Thus, an additional UTE sequence for better bone definition might not always be available.

Differences in the delineation of the body contour arose due to differences in the set-up between the CT and MR imaging. Despite acquiring data on the same day and using the same fixation devices, larger geometrical differences were found for the H&N patients. The potential impact of daily set-up variations between imaging sessions at these sites has already been evaluated in the literature [32], [33]. The mean average set-up error in any single dimension is reported to be up to 4 mm. In addition, MRI usually does not express a clear boundary which hinders the external contour delineation. For future studies, it will be crucial to identify voxels at the boundary that lie outside the patient to omit further interference while defining the patient outline and while performing the required registration processes for the atlas approach.

The last step of the evaluation consisted of comparing the dose distributions obtained from the sCT_bda_ and sCT_a_ with the CT dose distributions. For both the target and the OARs, both sCT-based dose distributions differed from the corresponding CT-based dose distribution, on average, no more than 1% of the original dose. These results are comparable to those already presented in the literature [8], [9], [11], [19], [23], [24]. Mean percentages of passing points within the external contour of 98–100% and 94–97% were achieved for both methods and cancer sites for the 3D local Ɣ-analyses with constraints of 3%_3 mm and 2%_2 mm, respectively. Ɣ-pass rates of the same order were reported by Korhonen et al. [11] (93–97% for 1%_1 mm 2D Ɣ-test), Jonsson et al. [15] (99% for 3%_3 mm Ɣ-test), Dowling et al. [19] (93–96% for 2%_2 mm 3D global Ɣ-test), Uh et al. [23] (98–99% for 2%_2 mm Ɣ-test), Andreasen et al. [24] (97% for 1%_1 mm 2D global Ɣ-test) and Siversson et al. [25] (99–100% for 2%_1 mm local Ɣ-test). Furthermore, dose distributions based on sCT_a_ showed a better PTV agreement and a more homogeneous gamma map with lower gamma values than sCT_bda_. As for the multi-atlas scheme a one-to-one estimation for each electron density voxel value is assigned, a greater similarity with the original dose distributions is expected. The magnitude of these dosimetric differences will also depend on the planning parameters (VMAT or multi-field plan), and on the geometry of the patient. In general, higher dosimetric differences were found for the H&N patients. These could be explained by the lack of MRI coverage and to the difficulties added by the large-scale postural changes between imaging sessions in the registration processes. Furthermore, these patients are more sensitive to dose errors as a mixture of bone, air and soft-tissue is present, while for prostate patients, the irradiated volume consists mostly of bone and soft-tissue.

The results of this feasibility study showed that both bulk density assignment and multi-atlas methods are suitable to perform dose calculations. Both approaches showed a good performance despite the limitations introduced by the suboptimal retrospective data: limited MRI FOV, the use of images from different scanners in the atlas and test population for the H&N patients and the presence of geometrical distortion within the MRI images.

As a result of the limited MRI FOV, large systematical differences within the beam path between the original CT and sCTs would be expected. To overcome this limitation, a density override approach assigning water equivalent density to all regions outside the MRI FOV but within the CT external contour was used for both CT and sCTs. This approach assures an evaluation as fair as possible, but in our opinion does not artificially improve the results as differences between CT and sCTs would only be related to electron density changes within the MRI FOV. In addition, for the H&N patients, the patient external outline was not fully covered in the MR images, which resulted in missing tissue at the back of the head and on the chin. These regions were assumed to be of air equivalent density. Filling these tissue gaps with water density could lead to results that could be better than in the true clinical situation whereas assuming an air density represents a ‘worst-case scenario’. Nevertheless, there is only missing tissue in a small number of slices (<10% of the sCT external volume) resulting in a minimal impact on the geometrical evaluation and a reduced effect on the dosimetric evaluation. This problem should be easy to overcome in the future when radiologists are aware that MR scans will also be used for RTP. Imaging protocols should be adapted for the FOV to cover the entire body contour and not only the PTV region.

Building a reliable atlas database is a pre-requisite to guarantee the good performance of this atlas-based approach. CT and MR images need to be acquired for a number of patients on the same day, under treatment position and using the same fixation devices. Ideally, all data should be collected using the same MR sequences and scanner, as MR intensities are highly dependent on the equipment. However, establishing scanner-specific atlases would be challenging or even unpractical considering the clinical reality. By testing our approach using data from different scanners, as for the H&N patients, we demonstrated the robustness of our method to these differences. Nevertheless, using as atlases images of patients acquired with the same sequence and on the same scanner as the test patient would improve the results.

MRI is also known to suffer from geometric distortions owing to the non-linearity of the imaging gradients over large fields of view. Spatial distortions in MR images vary with field strength and with the image acquisition protocol, which explains the difficulty to provide a general estimation on their magnitude. The development of correction techniques is a very active field of research in the MR community, and we expect the impact of these distortions in the context of photon radiotherapy to become insignificant in the future. A remark to consider is that patient-specific distortions due to magnetic susceptibility or imaging artifact in the MRI present a limitation for the generation of sCTs. For CT images, artifacts can be manually delineated, overwritten with appropriated density values and in this way corrected. Thus, for the sCT_bda_ approach they would not represent a restriction, but would compromise the performance of segmentation and density assignment. For an atlas-based approach, patient-specific abnormalities that are not represented in the atlas generation are a limitation and an exclusion criterion. However, this only concerns a limited number of patients.

Despite these limitations, a good dosimetric performance was achieved for both methods. However, the geometric evaluation urges caution. Bone and external delineations can only be performed automatically, and with a high degree of similarity with the planning CT, using the sCT_a_. For sCT_bda_, bone delineation has to be performed manually which is a very time consuming task, is subject to inter-observer variability, and is performed as a best guess, making this method unsuitable for clinical use. In contrast, the proposed atlas method automatically generates an sCT in around three hours without performance optimization. In the future, we suggest combining soft-tissue and target contours delineated directly on the MR image, with bone contours and HUs obtained from the proposed multi-atlas approach. Since the sCT_a_ is created in the same space as the MRI, the definition of the soft-tissue structures and target on the MRI can be easily propagated to the sCT_a_ for planning. As bony structures in the sCT_a_ images were shown to be consistent with the original CT, this image could also be used for patient positioning, at least for H&N patients where positioning relies on accurate bone geometry.

## 5 Conclusions

In this paper we evaluate two methods for MR-based RTP: the proposed multi-atlas CT synthesis method (sCT_a_) and a bulk density assignment method (sCT_bda_). Plans re-calculated on both sCT_a_ and sCT_bda_ showed an overall good dosimetric agreement with the clinical CT plan. However, only sCT_a_ can give an accurate bone delineation enabling patient positioning during treatment. Combining MR delineations with our multi-atlas scheme could improve the dosimetric and geometric accuracy of the treatment, and reduce the number of imaging procedures. Note that, due to the use of suboptimal-retrospective data, the results from this study should be interpreted as a conservative worst-case scenario.

Several methodological novelties were presented to guarantee the robustness of the proposed multi-atlas CT synthesis approach: (1) a robust affine was proposed to ensure the correct alignment between each atlas and the test patient; (2) the method was extended to handle multiple MR contrasts; (3) an iterative process was proposed to improve the synthesis accuracy in the bone region. To the best of our knowledge, this is the first time that a CT synthesis approach has been able to generate accurate sCT images for RT planning for both H&N and prostate patients.

## Figures and Tables

**Table 1:** MAE computed between the sCTs and planning CT images in three regions (in the external contour, in the bone region and the soft-tissue region within the MRI FOV) for both H&N and prostate patients. Mean and standard deviations (SDs) are shown along with range (in brackets).

		MAE (HU)			
Patient	ROI	sCT_bda_		sCT_a_	
		Mean ± SD	Range	Mean ± SD	Range
H&N	External	200.2 ± 23.4	[171.6;239.2]	90.7 ± 12.1	[80.5;113.8]
	Bone	553.6 ± 33.7	[518.4;611.1]	189.8 ± 16.3	[170.1;209.9]
	Soft-tissue	120.6 ± 17.2	[96.8;146.2]	68.1 ± 10.1	[57.9;84.8]
Prostate	External	85.2 ± 4.3	[79.3;92.7]	49.8 ± 4.6	[42.6;58.4]
	Bone	163.5 ± 9.2	[148.0;179.1]	119.7 ± 12.8	[102.1;147.9]
	Soft-tissue	49.8 ± 1.6	[46.3;52.0]	36.8 ± 4.7	[28.6;47.9]

**Table 2:** VI for the external and bone contours for both H&N and prostate patients. Mean and standard deviations (SDs) are shown along with range (in brackets).

		VI			
Patient	ROI	sCT_bda_		sCT_a_	
		Mean ± SD	Range	Mean ± SD	Range
H&N	External	1.03 ± 0.03	[1.02;1.06]	1.02 ± 0.03	[1.00;1.05]
	Bone	1.09 ± 0.04	[1.04;1.14]	0.96 ± 0.05	[0.90;1.02]
Prostate	External	1.01 ± 0.03	[1.00;1.06]	1.00 ± 0.02	[1.00;1.02]
	Bone	1.12 ± 0.04	[1.03;1.18]	0.99 ± 0.02	[0.95;1.00]

**Table 3:** DSC for the external and bone contours for both H&N and prostate patients. Mean and standard deviations (SDs) are shown along with range (in brackets).

		DSC			
Patient	ROI	sCT_bda_		sCT_a_	
		Mean ± SD	Range	Mean ± SD	Range
H&N	External	0.96 ± 0.01	[0.95;0.97]	0.98 ± 0.02	[0.96;0.99]
	Bone	0.78 ± 0.03	[0.72;0.83]	0.83 ± 0.03	[0.77;0.86]
Prostate	External	0.98 ± 0.02	[0.95;0.99]	0.99 ± 0.01	[0.98;0.99]
	Bone	0.85 ± 0.02	[0.80;0.89]	0.93 ± 0.01	[0.91;0.95]

**Table 4:** Percentage of passing points for the 3D local gamma test. Mean and standard deviations (SDs) are shown along with range (in brackets).

		Gamma Passing Rates (%)			
	Patient	sCT_bda_		sCT_a_	
		Mean ± SD	Range	Mean ± SD	Range
3%_3 mm	H&N	98.2 ± 0.6	[97.3;99.2]	98.4 ± 0.3	[98.0;98.3]
	Prostate	98.2 ± 1.0	[96.6;99.5]	99.8 ± 0.4	[99.0;99.8]
2%_2 mm	H&N	93.8 ± 1.1	[92.3;95.4]	94.0 ± 0.7	[93.0;95.3]
	Prostate	95.6 ± 1.5	[93.4;97.6]	97.1 ± 1.3	[95.4;99.0]

**Table 5:** PPD for the selected DVH points for H&N patients. Mean and standard deviations (SDs) are shown along with range (in brackets).

		CT – sCT percentage difference (%): H&N Patients			
ROI	DVH Point	sCT_bda_		sCT_a_	
		Mean ± SD	Range	Mean ± SD	Range
PTV	D98%	0.32 ± 0.85	[−0.79;1.96]	0.67 ± 0.62	[−0.19;1.78]
	Dmean	−0.21 ± 0.37	[−0.94;0.27]	−0.09 ± 0.33	[−0.60;0.23]
	D2%	−0.30 ± 0.34	[−0.92;0.06]	0.10 ± 0.29	[−0.36;0.40]
Right parotid	Dmean	−0.11 ± 0.49	[−1.07;0.47]	0.14 ± 0.43	[−0.64;0.68]
	D2%	−0.49 ± 0.63	[−1.48;0.56]	−0.04 ± 0.42	[−0.55;0.52]
Left parotid	Dmean	−0.09 ± 0.43	[−0.97;0.31]	0.08 ± 0.39	[−0.71;0.43]
	D2%	−0.52 ± 0.41	[−1.26;0.01]	−0.46 ± 0.62	[−1.45;0.37]
Spinal Cord	Dmean	−0.30 ± 0.20	[−0.68;−0.06]	0.01 ± 0.31	[−0.22;0.57]
	D2%	−0.22 ± 0.30	[−0.60;0.14]	−0.34 ± 0.37	[−0.88;0.19]

**Table 6:** PPD for the selected DVH points for prostate patients. Mean and standard deviations (SDs) are shown along with range (in brackets).

		CT – sCT percentage difference (%): Prostate Patients			
ROI	DVH Point	sCT_bda_		sCT_a_	
		Mean ± SD	Range	Mean ± SD	Range
PTV	D98%	0.72 ± 0.55	[0.02;2.14]	−0.39 ± 0.79	[−1.22;1.07]
	Dmean	0.43 ± 0.48	[−0.13;1.29]	−0.28 ± 0.67	[−1.54;1.07]
	D2%	0.43 ± 0.51	[−0.12;1.42]	−0.19 ± 0.67	[−1.06;1.40]
Bladder	Dmean	0.88 ± 0.32	[0.22;1.56]	−0.07 ± 0.72	[−1.17;2.04]
	D2%	0.75 ± 0.43	[0.17;1.45]	−0.36 ± 0.62	[−1.65;0.47]
Rectum	Dmean	0.61 ± 0.49	[−0.18;1.82]	−0.39 ± 0.90	[−1.74;1.57]
	D2%	0.21 ± 0.54	[−0.79;1.50]	−0.34 ± 0.46	[−0.89;0.53]

## Footnotes

2 http://cmic.cs.ucl.ac.uk/home/software.

3 http://plastimatch.org/plastimatch.html.
